# Unraveling the Enigma: Food Cobalamin Malabsorption and the Persistent Shadow of Cobalamin Deficiency

**DOI:** 10.3390/jcm14082550

**Published:** 2025-04-08

**Authors:** Emmanuel Andrès, Jean Edouard Terrade, María Belén Alonso Ortiz, Manuel Méndez-Bailón, Cosmina Ghiura, Chalène Habib, Thierry Lavigne, Xavier Jannot, Noel Lorenzo-Villalba

**Affiliations:** 1 Service de Médecine Interne, Hôpitaux Universitaires de Strasbourg, 67000 Strasbourg, France; jean-edouard.terrade@chru-strasbourg.fr (J.E.T.); cosmina-florina.ghiura@chru-strasbourg.fr (C.G.); charlene.habib@chru-strasbourg.fr (C.H.); xavier.jannot@chru-strasbourg.fr (X.J.); noellorenzo@gmail.com (N.L.-V.); 2 Servicio de Medicina Interna, Hospital Universitario de Gran Canaria Dr Negrin, 35010 Las Palmas de Gran Canaria, Spain; belalor@yahoo.es; 3 Servicio de Medicina Interna, Hospital Universitario Clínico San Carlos, 28040 Madrid, Spain; manuelmenba@hotmail.com; 4 Service d’Hygiène Hospitalière, Hôpitaux Universitaires de Strasbourg, 67000 Strasbourg, France; thierry.lavigne@chru-strasbourg.fr

**Keywords:** cobalamin deficiency, vitamin B12 deficiency, food cobalamin malabsorption, pernicious anemia, cobalamin treatment

## Abstract

Food cobalamin malabsorption (FCM) represents a prevalent, often underdiagnosed, etiology of vitamin B12 deficiency, particularly within an aging population. Unlike pernicious anemia, an autoimmune disorder targeting intrinsic factor, FCM stems from the impaired release of cobalamin from food proteins, primarily due to age-related gastric changes, medication-induced gastric hypochlorhydria, metformin, or non-immune atrophic gastritis. The clinical presentation of FCM mirrors that of general cobalamin deficiency, encompassing a spectrum of neurological (peripheral neuropathy, cognitive decline), hematological (megaloblastic anemia), and gastrointestinal (glossitis, anorexia) manifestations. Given the potential for irreversible neurological sequelae, early detection and intervention are paramount. High-dose oral cobalamin (125–250 mcg daily) has demonstrated efficacy, offering a convenient and cost-effective alternative to parenteral administration.

## 1. Introduction

Cobalamin (vitamin B12) is a vitamin found in fish, red meat, and dairy products. Vitamin B_12_ in animal food is bound to a protein which is subsequently broken down in the stomach by pepsin and hydrochloric acid to release free vitamin B_12_. This is afterwards bound to the R-protein (haptocorrin) found in saliva and gastric juice. From the enterohepatic circulation, the vitamin B12-R-protein complex is also secreted in bile. These complexes are then degraded by pancreatic enzymes to release free vitamin B12 in the duodenum. The free vitamin B12 is afterwards bound to intrinsic factor secreted by the gastric parietal cells, and then it travels undisturbed until the distal 80 cm of the ileum where it binds to mucosal cell receptors. Subsequently, vitamin B12 is carried by the transport protein transcobalamin via the portal system to all cells in the body for utilization. About 60% of vitamin B12 from food is absorbed through this pathway, and any pathophysiological changes in the stomach, pancreas, and intestine result in the disturbance of vitamin B12 absorption [1].

Cobalamin (vitamin B12) deficiency, a seemingly simple nutritional deficit, continues to challenge clinicians with its diverse clinical presentations and the complexities of its underlying etiologies [2]. While pernicious anemia (PA, also named Biermer’s disease), an autoimmune disorder, has long been recognized as a primary cause, the often-overlooked condition of food cobalamin malabsorption (FCM) deserves renewed attention [3].

FCM was first described by Carmel in 1988 [4]. FCM is a condition where the body cannot effectively extract vitamin B12 from food, leading to deficiency despite adequate dietary intake [5]. The primary problem is an inability to release vitamin B12 from dietary proteins. Thus, affected individuals can absorb vitamin B12 supplements in which the vitamin is not protein-bound, but they are less able to absorb dietary vitamin B12 bound to food proteins due to conditions that interfere with dissociation of the vitamin from food proteins [6].

## 2. Methods

We conducted a review on food cobalamin malabsorption syndrome. A comprehensive search was performed in the PubMed, Embase, and Cochrane Library databases up to March 2025. Keywords used included “food-cobalamin malabsorption”, “cobalamin deficiency”, and “oral cobalamin therapy”. Single case studies and unpublished works in English or French were excluded. The bibliographic references of selected articles were also examined to identify additional studies. Discrepancies were resolved by consensus or consultation with a third researcher. This methodology aims to provide a reliable synthesis of the state of the art concerning food cobalamin malabsorption.

## 3. Epidemiology

FCM represents a significant yet frequently underdiagnosed cause of vitamin B12 deficiency, particularly in the elderly population. Prevalence of vitamin B12 deficiency in elderly patients was 40.5% compared with 17.5% in control subjects [7]. Mild and/or subclinical vitamin B12 deficiency has been documented at relatively high frequency in older adults, with various observational studies describing a prevalence of approximately 5 to 20% depending on the population studied and the laboratory criteria used to define deficiency [7,8,9,10]. Other studies indicate that up to 60% of individuals over 65 with vitamin B12 deficiency may have FCM [11]. Otherwise, FCM has been reported to cause vitamin B12 deficiency in 40% to 70% of individuals aged 65 and above [4,12,13].

The median patient age was 66 and 67 years in two studies of 80 and 201 patients, respectively, with a predominancy of female patients [13,14].

## 4. Pathophysiology

PA and FCM differ in pathophysiology. PA is an autominumme autoimmune disorder characterized by the destruction of gastric parietal cells (autoimmune atrophic gastritis), leading to intrinsic factor deficiency and impaired vitamin B_12_ absorption. The immune system can also produce antibodies that directly target intrinsic factor, preventing it from binding to vitamin B12. Intrinsic factor is essential for vitamin B_12_ absorption in the terminal ileum. The lack of intrinsic factor leads to vitamin B_12_ malabsorption, resulting in megaloblastic anemia and potential neurological complications.

**Figure 1:**
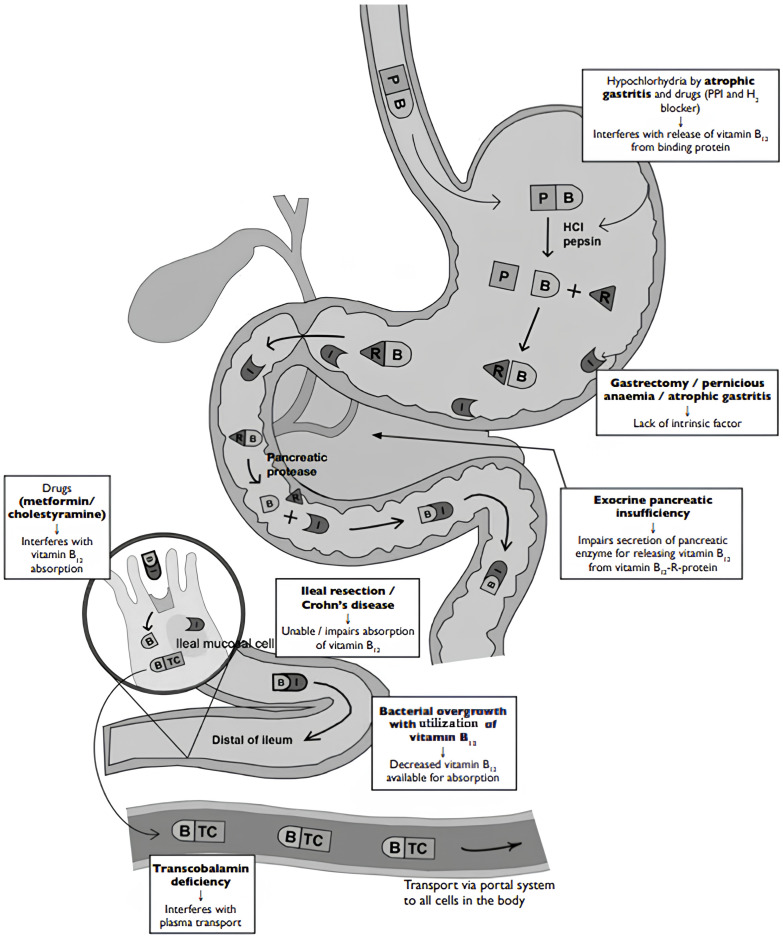
Pathophysiology of pernicious anemia (Biermer’s disease) and food cobalamin malabsorption [13]. **Legend:** B: vitamin B12, HCI: hydrochloric acid, I: intrinsic factor, P: animal protein, PPI: proton pump inhibitor, R: protein, TC: transcobalamin.

Unlike PA, FCM stems from gastric achlorhydria or non-immune atrophic gastritis, leading to impaired proteolytic digestion of food-bound cobalamin (Figure 1) despite normal intrinsic factor production. Pancreatic dysfunction can also lead to FCM. Conditions such as chronic gastritis, long-term use of acid-suppressing medications, or pancreatic insufficiency lead to the inadequate release of vitamin B_12_ from food proteins.

Despite normal intrinsic factor production, vitamin B_12_ remains bound to food proteins and is not absorbed in the ileum. This malabsorption can cause vitamin B_12_ deficiency, leading to the same symptoms as PA. This distinction is important, as the diagnostic and therapeutic approaches differ significantly [15].

## 5. Etiology

Currently, the primary causes or associated conditions/factors of FCM mainly include advancing age, gastric disorders, *Helicobacter pylori* infection, and medications such as proton pump inhibitors (PPIs) and metformin (Table 1) [13,14]. A study of 80 internal medicine patients revealed that gastric atrophy was by far the most frequent cause (39%), followed by Helicobacter pylori infection (12.2%). Alcohol misuse and chronic intake of biguanides or PPIs/H2 receptor antagonists were associated with 13.7%, 10%, and 7.5%, respectively. Rarer causes such as systemic diseases (Sjögren’s syndrome and scleroderma) and pancreatic insufficiency were found in 5% and 3.8%. Finally, 12.5% of cases were idiopathic or age-related [14].

## 6. Clinical Manifestations

The clinical consequences of FCM can be insidious, mirroring those of other causes of cobalamin deficiency. However, patients with FCM may present with plain clinical and hematological manifestations, often masked by age-related comorbidities. Many individuals with food cobalamin malabsorption are not anemic and do not have other features of megaloblastic anemia, such as macrocytosis or hypersegmented neutrophils, sometimes referred to as subclinical deficiency (Table 2) [13,14,18].

Because FCM can develop slowly, the symptoms may be very gradual and easily overlooked (subtle clinical manifestations) [4,19]. The milder symptoms seen in some patients can result from an IF-dependent transport mechanism that is still functioning when the hydrochloric acid (HCl) production is reduced.

Subtle neurological manifestations include mild paresthesia; slight sense of unsteadiness, particularly in low-light conditions or when walking on uneven surfaces; minor cognitive changes; and mood alterations with increased irritability, mild depression, or subtle changes in mood that may not be readily recognized as a medical issue [20]. Early detection and treatment are important to prevent irreversible neurological damage. Neurological manifestations are most frequent in internal medicine patients [14,20].

Subtle hematological manifestations have also been reported as mild fatigue, slight pallor, and occasional weakness [4,19]. In this context, occasional mild loss of appetite and minor digestive discomfort are noticed. In elderly populations, these subtle changes can significantly impact quality of life. Hematological manifestations, including macrocytic anemia, may be less pronounced or even absent, further complicating the diagnostic process. Other cytopenias may be found in these patients, but the discovery of a bicytopenia/pancytopenia should lead to a bone marrow aspiration or osteomedullary biopsy performance, especially in subjects over 65 years of age with no suggestive clinical context, in search of a hemopathy. Bone marrow aspiration or osteomedullary biopsy may be proposed depending on practice and country but always in conjunction with a molecular biology investigation (5q- syndrome, etc.) and a karyotype. These investigations will allow clinicians to avoid missing a myelodysplastic syndrome, which may be fortuitously associated with a B12 deficiency [14,20].

These subtle symptoms are often dismissed as normal aging or stress, leading to delayed diagnosis. Thus, it is important for clinicians to maintain a high index of suspicion for cobalamin deficiency, especially in elderly patients presenting with these vague symptoms [21].

Neuropsychiatric abnormalities can occur even in the absence of significant hematological changes [14,20]. Patients may also present no symptoms or clinical signs, 30% in our study [20].

## 7. Diagnosis

The diagnostic pathway for FCM requires a high index of suspicion [6,11]. While serum cobalamin levels remain the initial screening tool, they may not always accurately reflect tissue cobalamin status. Assessing methylmalonic acid (MMA) and homocysteine levels can provide valuable insights into metabolic cobalamin deficiency [22]. Importantly, intrinsic factor antibodies are typically absent, differentiating FCM from PA [5,6]. Furthermore, the Schilling test, although less frequently performed today, can aid in differentiating FCM from PA.

The Schilling test, introduced in 1953, has historically been considered the gold standard for diagnosing FCM [4]. This test involves administering a radiolabeled dose of vitamin B12 and measuring its excretion in the urine to assess absorption efficiency. However, the Schilling test and its modified versions are no longer widely available in clinical practice. Consequently, diagnosing FCM often relies on a combination of clinical evaluation, laboratory findings indicating vitamin B12 deficiency, and the exclusion of other potential causes of malabsorption [6].

While the Schilling test was once the definitive diagnostic tool for FCM, its current unavailability necessitates a comprehensive approach that integrates patient history, clinical symptoms, and laboratory assessments to accurately diagnose FCM (Table 3) [3,4,6,23].

## 8. Treatment

The therapeutic approach to FCM differs from that of PA [24,25,26,27,28,29,30]. Traditionally, vitamin B12 deficiency has been treated with intramuscular injections because of the probability of low absorption in some conditions [29]. Treatment regimens are not based on robust studies and differ from center to center and country to country, but it would seem preferable to progressively increase the interval between two injections [26,27].

However, research indicates that oral cobalamin therapy is an effective alternative for managing FCM. Oral cobalamin supplementation, often at higher doses (650 µg per day), is generally effective in replenishing cobalamin stores [30]. Unlike PA, which typically requires lifelong parenteral cobalamin, oral supplementation can be a practical and cost-effective option for patients with FCM. If the symptoms are acute and potentially serious, the intramuscular form is often preferred by the majority of practitioners. The oral form is just as effective as the IM form if therapeutic compliance is good, and if the efficacy of treatment in terms of vitamin B12 and normalization of total homocysteine is monitored, even in severe forms. It may be necessary to switch from oral to intramuscular and vice versa depending on the patient’s response [31].

Multiple studies have demonstrated the effectiveness of oral vitamin B12 in treating FCM (Table 4) [21]. A systematic review showed that oral cobalamin therapy adequately corrects cobalamin deficiency in patients with FCM. In our experience, oral supplementation of FCM patients achieved satisfactory hemoglobin and vitamin B12 levels (11.5 g/dL + 1.9 g/dL over 3 months), with higher figures for IM treatment (13.2 g/dL + 3.9 g/dL) [14].

Research suggests that a daily dose of approximately 250 micrograms of oral cyanocobalamin is sufficient for managing FCM [24,32]. Regular monitoring of vitamin B12 levels is essential to ensure therapeutic efficacy, check compliance with oral treatment, and adjust dosages as needed. While oral therapy is effective for many, individual absorption rates may vary [24]. Some patients might still require intramuscular injections, especially if they have conditions affecting intestinal absorption [2].

## 9. Conclusions

FCM is a common, yet commonly missed, disorder that may be masked by many other conditions. The challenge lies in the under-recognition of FCM. The subtle clinical presentation, the overlap with age-related conditions, and the potential for normal or borderline serum cobalamin levels can lead to diagnostic delays. Increased awareness among clinicians regarding the prevalence and clinical manifestations of FCM is important as well as iatrogenic causes such as PPI treatment.

## Figures and Tables

**Table 1:** Causes or associated conditions/factors of food cobalamin malabsorption [13,14,15,16,17].

Cause	Mechanism	Associated Conditions/Factors
Atrophic gastritis	Reduced secretion of hydrochloric acid (HCl) and pepsin, impairing the release of cobalamin from food proteins.	Aging, *Helicobacter pylori* infection, autoimmune gastritis
*Helicobacter pylori* infection	Inflammation of the gastric mucosa, potentially leading to atrophic gastritis and reduced acid production.	Chronic *H. pylori* infection
Proton pump inhibitors (PPIs)	Suppression of gastric acid secretion, hindering the release of cobalamin.	Long-term PPI use
H2 receptor antagonists	Reduction in gastric acid secretion, similar to PPIs, but generally to a lesser degree.	Long-term H2 blocker use
Gastric surgery (partial gastrectomy)	Removal of parietal cells, which produce HCl and intrinsic factor, affecting cobalamin release and absorption.	Post-surgical conditions
Pancreatic insufficiency (less common)	Reduced secretion of pancreatic enzymes, which play a role in cobalamin release in the small intestine.	Chronic pancreatitis, cystic fibrosis, related to alcohol misuse
Systemic disease	In Sjögren syndrome, loss of gastric acidity caused by lymphocytic gastric infiltration and altered production of salivary carrier protein (haptocorrin) might explain B12 deficiency. Multifactorial in systemic scleroderma: gastro intestinal involvement and narrowed oral orifice, low salivary secretation, depression, and drug intake.	Sjögren syndrome, systemic sclerodermia
Aging	General decline in gastric acid production.	Elderly populations

**Table 2:** Clinical and hematological manifestations related to cobalamin deficiency [13,14,18].

Category	Manifestation	Description	Frequency
Hematological	Megaloblastic anemia	Characterized by large, immature red blood cells (aregenerative macrocytic anemia)	Frequent
	Increased Mean Corpuscular Volume (MCV) *	Elevated average volume of red blood cells	Frequent
	Hypersegmented neutrophils	Neutrophils with an increased number of lobes in their nucleus	Frequent
	Thrombocytopenia, neutropenia, pancytopenia, thrombotic microangiopathy	Bone marrow aplasia	Rare
Neurological	Peripheral neuropathy	Tingling, numbness, and pain in the extremities	Frequent
	Subacute combined degeneration of the spinal cord	Damage to the spinal cord, leading to gait disturbances, loss of balance, and weakness	Classic
	Cognitive impairment	Memory loss, confusion, and difficulty concentrating	Unknown
	Optic neuropathy	Visual loss	Rare
Neuropsychiatric	Depression	Persistent feelings of sadness and hopelessness	Unknown
	Irritability	Increased agitation	Unknown
	Psychosis	In severe cases	Unknown
Gastrointestinal	Glossitis (de Hunter)	Inflammation of the tongue	Classic
	Loss of appetite	Reduced desire to eat	Frequent
	Constipation or diarrhea	Changes in bowel habits	Unknown
	Jaundice	Hemolysis	Classic
General	Fatigue	Persistent tiredness and weakness, linked to anemia	Frequent
	Pallor	Pale skin linked to anemia	Frequent
	Weakness	General muscle weakness linked to anemia	Frequent

* Macrocytosis is optional, because patients may present with concomitant B12 and iron deficiency and normocytic anemia. Also, patients first develop macrocytosis before developing overt anemia.

**Table 3:** Diagnostic criteria of food cobalamin malabsorption [3,4,6,23].

Category	Diagnostic Criteria	Description
Clinical presentation	Symptoms of cobalamin deficiency	Includes neurological symptoms (peripheral neuropathy, cognitive changes), fatigue/pallor, and gastrointestinal symptoms (glossitis).
Exclusion of nutritional deficieny	Vegetarian or vegan diet	Vitamin B12 is found exclusively in animal-derived foods such as meat, fish, dairy, and eggs.
Exclusion of other malabsorption syndromes	Tests to rule out other causes	It is important to rule out other causes of malabsorption, like celiac disease, or small intestinal bacterial overgrowth, and any history of digestive tract surgery.
Iatrogenic condition	Current medication	It is important to verify any possible medication leading to FCM.
Laboratory findings	Low serum cobalamin (vitamin B12) levels	Indicates a deficiency. Serum B12 levels have a poor sensitivity and specificity.
	Elevated methylmalonic acid (MMA) and/or homocysteine levels	Confirms cobalamin deficiency at a metabolic level. MMA can be measured in blood and urine; homocysteine levels have to be measured after fasting; MMA is very specific and sensitive in B12 deficiency, while homocysteine can be elevated in folate/pyridoxine deficiency or hypothyroidism; other causes of anemia (if present) should be tested for (folate deficiency, iron deficiency).
	CobaSorb test	CobaSorb is based on the measurement of holoTC in a blood sample collected before and after oral intake of a high physiological dose of vitamin B12.
	Normal intrinsic factor antibodies	This test is very important to differentiate this condition from pernicious anemia. Because pernicious anemia is an autoimmune condition that attacks the intrinsic factor, these antibodies will be present. In food cobalamin malabsorption, they are not present.

**Table 4:** Cobalamin treatment: oral vs. parenteral [2,24,26].

Feature	Food Cobalamin Malabsorption	Pernicious Anemia
Underlying cause	Impaired release of cobalamin from food proteins due to gastric dysfunction (e.g., atrophic gastritis)	Autoimmune destruction of parietal cells, leading to a lack of intrinsic factor, which is essential for cobalamin absorption in the ileum
Oral cobalamin treatment	* High-dose oral cobalamin can be effective. The high dose allows for some absorption via passive diffusion, bypassing the need for intrinsic factor * Typical oral doses range from at least 125 to 250 mcg per day	* High-dose oral cobalamin can also be effective, due to the passive diffusion of the vitamin * Typical oral doses range from at least 1000 to 2000 mcg daily
Parenteral cobalamin treatment	* Effective, especially in cases of severe deficiency or neurological symptoms * Intramuscular injections of cobalamin (e.g., cyano- or hydroxocobalamin) are commonly used	* Traditionally, parenteral cobalamin (intramuscular injections) has been the standard treatment * This ensures reliable delivery of cobalamin, bypassing the impaired absorption
Considerations	* Oral therapy is often preferred for its convenience and cost-effectiveness * Monitoring of cobalamin levels is essential to ensure adequate treatment response	* While oral therapy is effective, some clinicians may prefer initial parenteral therapy for rapid repletion, especially with neurological symptoms * Lifelong treatment is required
Typical parenteral dosing	Cyano- or hydroxocobalamin 1000 mcg intramuscularly, typically once per week for several weeks (at least 12 weeks), then monthly	Cyano- or hydroxocobalamin 1000 mcg intramuscularly, typically once par day for one week, then weekly for several weeks (at least 4 weeks), then monthly
